# A Multi-Component and Multi-Functional Synergistic System for Efficient Viscosity Reduction of Extra-Heavy Oil

**DOI:** 10.3390/molecules30224446

**Published:** 2025-11-18

**Authors:** Zuguo Yang, Yanxia Liu, Jing Jiang, Lijuan Pan, Dandi Wei, Xingen Feng, Long He, Jixiang Guo, Yagang Zhang

**Affiliations:** 1 Petroleum Engineering Technology Research Institute, Northwest Oilfield Company, Sinopec, Urumqi 830011, China; yangzg.xbsj@sinopec.com (Z.Y.); panlij.xbsj@sinopec.com (L.P.); weidd6099.xbsj@sinopec.com (D.W.); fengxg9687.xbsj@sinopec.com (X.F.); 2 School of Materials and Energy, University of Electronic Science and Technology of China, Chengdu 611731, China; liuyanxia100@uestc.edu.cn (Y.L.); 202321030310@std.uestc.edu.cn (J.J.); 3 Key Laboratory of Enhanced Recovery for Fracture-Cave Oil Reservoir, Sinopec, Urumqi 830011, China; 4 Unconventional Petroleum Research Institute, China University of Petroleum, Beijing 102249, China

**Keywords:** extra-heavy oil, blending and viscosity reduction, washing oil, surfactants, aromatic compound, multi-functional

## Abstract

The extra-heavy oil in the Tahe Oilfield of China has extremely high viscosity, as it is rich in the heavy components asphaltene and resin, creating significant difficulties in its exploitation and transportation. Therefore, it is important to effectively reduce the viscosity and improve the fluidity of this extra-heavy oil. The traditional viscosity reduction method suffers from a high blending ratio and a shortage of light crude oil resources for extra-heavy oil blending. In this study, coal tar and washing oil—widely available low-cost by-products of the coal chemical industry—are used for extra-heavy oil blending and viscosity reduction. Washing oil—containing light components distilled from coal tar—was highly effective in reducing the viscosity of extra-heavy oil. When the dilution ratio of washing oil is 0.25, the viscosity of extra-heavy oil is reduced to 1214 mPa·s, and the viscosity reduction rate is 99.8%, indicating that washing oil is an efficient viscosity-reducing agent in extra-heavy oil blending. GC-MS showed that the washing oil contained abundant aromatic hydrocarbons and aromatic heterocyclic rings. A multi-component viscosity reduction system using washing oil coupled with toluene, xylene, and surfactant achieved an even better viscosity reduction effect. In conclusion, we designed a low-cost, high-efficiency, multi-component, and multi-functional synergistic system for extra-heavy oil viscosity reduction in the Tahe Oilfield. In the proposed working mechanism, aromatic hydrocarbons and aromatic heterocyclic rings in washing oil can intercalate into the layered structure of dense asphaltene aggregates, thereby dispersing and dissociating them.

## 1. Introduction

Heavy oil occupies a large proportion of the world’s oil and gas resources [1]. China’s heavy oil resources amount to about 19.87 billion tons, of which 3.55 billion tons have been verified, and the development potential is enormous. Compared with ordinary light crude oil, heavy oil has [2] high viscosity, high density, and high asphaltene and resin content [3]. The heteroatom content—such as O, S, and N and metal elements such as Fe, Ni, and V—in heavy oil is also quite high [4,5]. Poor fluidity during storage and transportation has created significant difficulties for the extraction of heavy oil, so reducing its viscosity is extremely important [6]. Extra-heavy oil is the most viscous part of heavy oil resources, and its viscosity is greater than 5 × 10^5^ mPa·s at 50 °C [7]. In western China, the Tarim Oilfield, Lunnan Oilfield, and Tahe Oilfield are rich in heavy oil resources, especially extra-heavy oil. The reservoir is buried very deep and has a high temperature, high pressure, high salinity, and extremely high viscosity. Viscosity reduction for extra-heavy oil is urgently needed considering national energy security [8]. Therefore, it is important to develop low-cost and high-efficiency viscosity-reducing agents for extra-heavy oil. However, problems such as resource shortages, high prices, and large dosages exist in using dilute oil or diesel oil to reduce oil viscosity. Nonetheless, the by-products of the coal chemical industry, coal tar and washing oil, have large outputs and low costs, and are some of the most promising candidates for replacing light crude oil and diesel oil in extra-heavy oil viscosity reduction.

**Figure 1:**
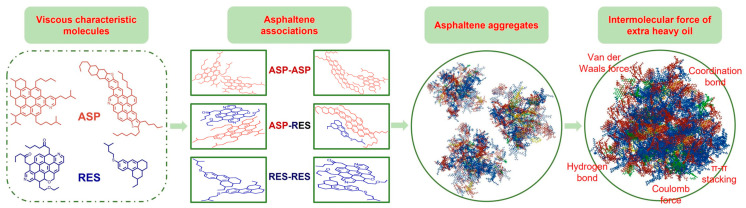
The formation mechanism of high-viscosity extra-heavy oil.

Figure 1 shows the major interactions and formation process of highly viscous extra-heavy oil. Heavy oil contains large amounts of asphaltene, Ni, V, O, N, S, and other elements [9,10]. The π-π stacking between thick aromatic hydrocarbons, hydrogen bonding between heteroatoms, and complexation caused by heavy metal ions in the molecular structure form dense layered asphaltene, which aggregates into highly dense asphaltene [11,12]. Asphaltene is formed through aggregation and entangling, and the aggregates further form a strong viscoelastic resin system with a three-dimensional network structure, resulting in a sharp increase in viscosity. π-π stacking and hydrogen bonding also contribute as the thickening factors [13]. Another important factor affecting the viscosity of heavy oil is wax crystals, which affect the flow performance of crude oil at low temperatures [14]. At temperatures above the wax evolution point, the wax dissolves in the crude oil. When the temperature is lower than the wax evolution point but higher than the freezing point, the wax gradually crystallizes out, and the viscosity of crude oil increases significantly because of the strong interaction between the wax crystals. If the temperature is lower than the freezing point, the wax crystals will further connect to form a 3D network structure, and the crude oil will lose its fluidity.

This study investigates extra heavy oil from the Tahe Oilfield in western China and develops a multi-component functional synergistic viscosity reduction system. The system utilizes coal tar/washing oil as the primary components, supplemented with small-molecule aromatic compounds and surfactants. A systematic investigation was conducted to compare the viscosity reduction performance of coal tar, washing oil, light crude oil, and diesel oil when blended with extra heavy oil. The optimal blending ratio between washing oil and extra heavy oil was determined. Furthermore, the synergistic effects of multi-component systems caused by combining washing oil with aromatic compounds (toluene; xylene) and surfactants (SDBS; OP-10) were thoroughly investigated and discussed. This research provides a promising new approach for the viscosity reduction of extra heavy oil in the Tahe Oilfield.

## 2. Results and Discussion

### 2.1. Viscosity Reduction Effect of Blending Coal Tar

Coal tar is a viscous liquid obtained during the distillation (isolation through air heating) and gasification of coal [15,16]. Coal tar contains many aromatic components [17]. These can be embedded into the interlayer of densely packed structures formed via π-π stacking, disrupt intermolecular interactions within asphalt, and reduce the viscosity of heavy oil and improve its fluidity. N, O, and S in heterocycles can effectively break the crosslinking points formed by charge action in asphalt [18]. Therefore, theoretically, coal tar is a promising candidate for blending and reducing the viscosity of extra-heavy oil.

The extraction of heavy oil from the Tahe Oilfield requires that the viscosity of the diluted mixed oil be less than 2000 mPa·s at 50 °C. The experimental temperature was 50 °C, and the viscosity values of the extra-heavy oil and coal tar were 520,350 and 1834 mPa·s, respectively. Different viscosities and viscosity reduction rates were obtained by mixing different ratios of coal tar and extra-heavy oil (Table 1).

Table 1 shows the viscosity reduction performance of coal tar. When the dilution ratio is 2, the viscosity of the mixed oil after dilution is 4283 mPa·s, and the viscosity reduction rate is 99.18%. When the dilution ratio is increased to 5, the viscosity of the mixed oil is reduced to 1900 mPa·s, and the viscosity reduction rate is 99.63%, meeting the requirements of the oilfield (<2000 mPa·s). These results demonstrate that coal tar can greatly reduce the viscosity of extra-heavy oil. However, due to the high viscosity of coal tar at 50 °C (1834 mPa·s), the dilution ratio is quite high, meaning that the required amount is very large; this not only increases the cost of extra-heavy oil exploitation but also is not viable for on-site field use.

The by-product of coal tar can be distilled as washing oil, another viscosity reduction candidate. Thus, the blending and viscosity reduction effects of washing oil were further investigated.

### 2.2. Viscosity Reduction Effect of Blending Washing Oil

Washing oil is the light component of coal tar distillation (230~300 °C distillate) [19], accounting for about 4.5~10% of the total, and China’s annual output is more than 1 million tons. Washing oil mainly comprises naphthalene, acenaphthene, fluorene, oxyfluorene, phenol, and azaryl ring compounds. Washing oil is rich in aromatic groups and has good oil solubility, which can effectively penetrate the asphaltene and resin of heavy oil. It can distort the lattice structure of wax crystals and aromatic lamellae in asphaltene aggregation, leading to anti-wax and anti-condensation effects. In addition, the hydrogen bond receptors of washing oil’s chemical components can compete for the hydrogen bonds of the original asphaltene, disintegrating the original hydrogen bonds. This results in the internal disassociation of some of the asphaltene aggregates; thus, the molecular weight decreases, and the viscosity of the heavy oil is reduced. Washing oil has a raw material cost of approximately 4000 CNY/ton, whereas conventional diesel oil is priced at around 7000 CNY/ton. Notably, both reagents incur comparable logistics expenses, with transportation costs estimated at 160 CNY/ton, given their local sourcing within a 200 km radius of the Tahe Oilfield. The former is much cheaper than the latter. Therefore, using washing oil to develop a new high-performance, low-cost, high-efficiency viscosity reducer is highly desirable for the development of extra-heavy oil resources in the Tahe Oilfield.

**Figure 2:**
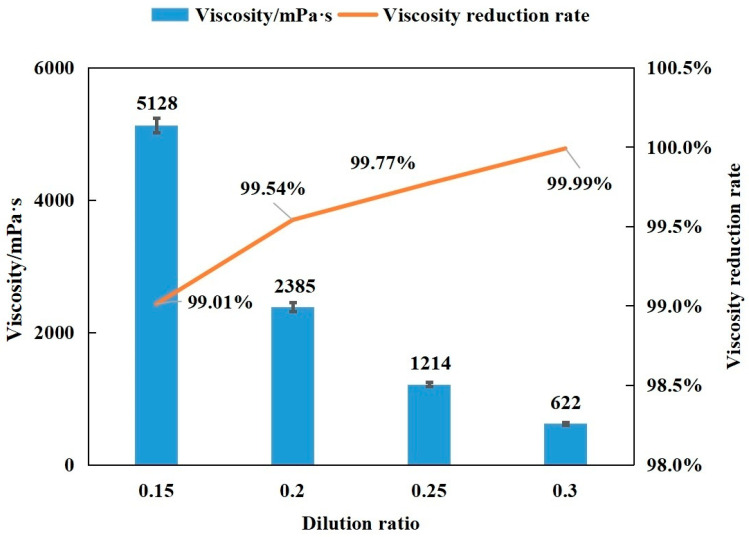
The viscosity reduction effect of washing oil on extra-heavy oil (50 °C).

At 50 °C, the viscosity of extra-heavy oil is 520,350 mPa·s, but the viscosity of washing oil is less than 10 mPa·s. The viscosity reduction effects of different mixtures were determined by mixing different proportions of washing oil into extra-heavy oil (see Figure 2 and Table 2).

The results showed that at 50 °C, the dilution ratio of washing oil increased from 0.15 to 0.3, the viscosity of mixed oil rapidly decreased from 5128 to 622 mPa·s, and the viscosity reduction rate increased from 99.01% to 99.99%. When the dilution ratio of washing oil was 0.25, the viscosity of the mixed oil was reduced to 1214 mPa·s, which was better than that of coal tar (coal tar–extra-heavy oil = 5:1; viscosity: 1900 mPa·s). This finding confirms that washing oil is an efficient extra-heavy oil blending viscosity reducer, with less dilution and an excellent viscosity-reducing effect.

**Figure 3:**
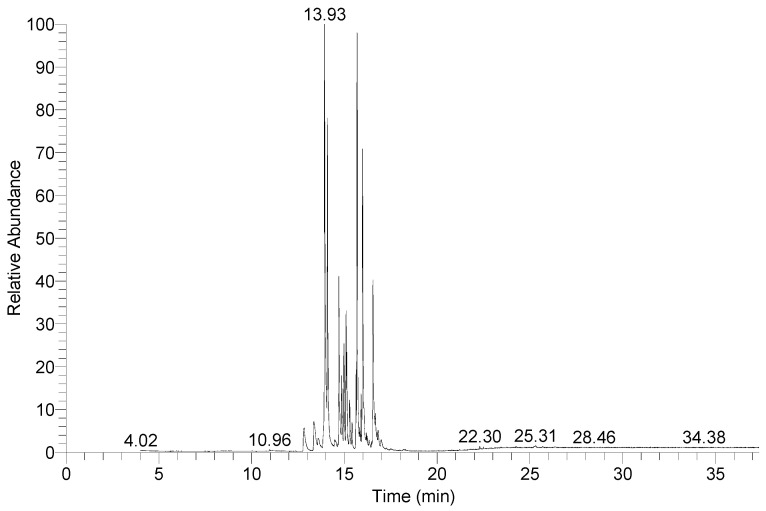
Total ion chromatogram of washing oil from GC-MS analysis.

**Figure 4:**
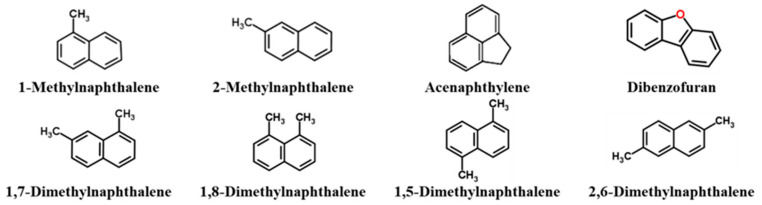
Structural formula of main chemical components of washing oil.

To explore its viscosity reduction mechanism, the chemical composition of washing oil was analyzed. The washing oil contained abundant aromatic hydrocarbons. A total of 32 components were detected using GC-MS (Figure 3, Table S1), and the main components were naphthalene and substituted naphthalene compounds (Figure 4). The content of the most abundant component, monomethylnaphthalene, was 31.82%, and there were two isomers, 1-methylnaphthalene and 2-methylnaphthalene, whose contents were 20.52% and 11.30%, respectively. Acenaphthene content ranked second with 16.39%. The dimethylnaphthalene content was 12.76%, and four isomers, 1, 7-dimethylnaphthalene, 1, 8-dimethylnaphthalene, 1, 5-dimethylnaphthalene, and 2, 6-dimethylnaphthalene, at 3.84%, 6.23%, 1.89%, and 0.80%, respectively. The dibenzofuran content was fourth at 12.58%. The aromatic groups of these compounds in washing oil can destroy wax crystals and the aromatic lamellar lattice structure of asphaltene aggregates [18], internally disintegrating them; the molecular weight becomes smaller, and the viscosity of heavy oil is reduced.

**Figure 5:**
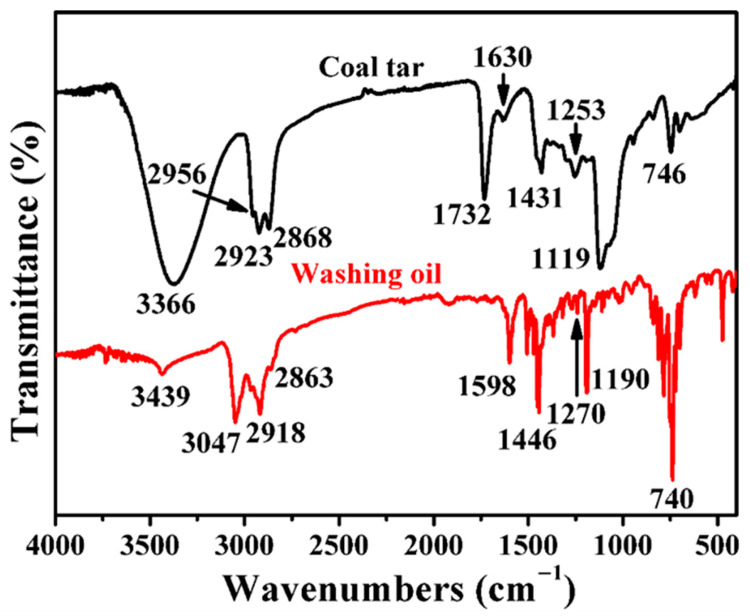
FT-IR spectra of coal tar and washing oil.

The FT-IR spectra of the separated components of coal tar and washing oil were measured, and the results are presented in Figure 5, where the absorption peaks of aliphatic CH_3_- and -CH_2_- asymmetric stretching vibrations (2923 and 2918 cm^−1^) and symmetric stretching vibrations (2868 and 2863 cm^−1^) appear. The absorption peaks of in-plane bending vibrations (1431 and 1446 cm^−1^) were also obtained [11,20]. The absorption peaks of coal tar and washing oil at 746 and 740 cm^−1^ were caused by the oscillating vibration of -(CH_2_) _n_-; the amount of adjacent -CH_2_- is more than four, indicating the presence of a long-chain hydrocarbon structure [21]. The stretching vibration absorption peaks of aromatic C-H appeared at 2956 and 3047 cm^−1^ for coal tar and washing oil, and the stretching vibration peaks of the aromatic C=C skeleton appeared at 1630 and 1598 cm^−1^, respectively, indicating the existence of aromatic groups.

The absorption peak strength of the oxygen-containing groups in coal tar is stronger than that of washing oil. The absorption peaks of coal tar at 3366, 1253, and 1119 cm^−1^ and washing oil at 3439, 1270, and 1190 cm^−1^ are due to the C-O vibrations of alcohols, phenols, and ethers, respectively. The stretching vibration peak of C=O of aromatic anhydride appears at 1732 cm^−1^ in coal tar, which can be attributed to the high amount of asphaltene and resin. The lone electron pair of oxygen in C=O has strong polarity and easily concentrates in asphaltene and resin containing more aromatic benzene rings [22], which leads to the high viscosity of coal tar and a poor viscosity reduction effect.

The results show that washing oil is an excellent viscosity reduction agent, and its working mechanism is proposed as follows. (a) The aromatic components of oil washing can act like a nail. This nail can penetrate the asphaltene layer and break the π-π interaction in the asphaltene aggregate, reducing the viscosity of extra-heavy oil. (b) The number of light components in washing oil is high, making it an effective solvent and reducing the concentration of asphaltenes in extra-heavy oil. (c) The alkane components of washing oil can act like dilute media to weaken the interaction between asphaltene macromolecules and prevent the formation of the spatial network structure of extra-heavy oil.

Only a small amount of washing oil is required for viscosity reduction, and it is highly efficient (Figure 2, Table 2). Moreover, the organochlorine content in washing oil is 0.0055%, which meets the requirements for on-site field use (<0.05%).

To further improve its viscosity-reducing performance and efficiency on extra-heavy oil, washing oil was combined with other components to develop a multi-component, multi-functional synergistic system.

### 2.3. Viscosity Reduction Effect of Blending Washing Oil/Light Crude Oil

Light crude oil is a commonly used blending viscosity reducer in oilfield exploitation and transportation, and it has a good viscosity reduction effect on extra-heavy oil. However, with the large-scale development of oilfields, light crude oil resources are very limited, and this shortage is becoming a major challenge. Therefore, in practical applications, to reduce the dilution ratio of light crude oil, washing oil with a good viscosity reduction effect can be mixed with light crude oil to partially replace it. At 50 °C, the viscosities of extra-heavy oil, washing oil, and light crude oil are 520, 350, 10, and 220 mPa·s, respectively. The viscosity reduction effects of washing oil/light crude oil were investigated separately. To this end, washing oil was used to partially replace dilute oil as the dilute mixing medium (Table 3), and washing oil was used as an additive to light crude oil (Table 4).

Table 3 shows that when the dilution ratio of light crude oil is 1.4, the viscosity of the mixed oil is 1838 mPa·s at 50 °C, and the viscosity reduction rate is 99.65%, meeting the viscosity reduction standard (viscosity at 50 °C is less than 2000 mPa·s). When washing oil replaces partial light crude oil as a dilute mixing medium, the dilution ratio is reduced to 0.3~0.7, the viscosity of the mixed oil is 1409~2097 mPa·s, and the viscosity reduction rate is greater than 99.60%. In particular, when the dilution ratio of the blending medium is 0.3 (washing oil–light crude oil–extra-heavy oil = 0.2:0.1:1), the viscosity of the mixed oil is 1775 mPa·s, and the viscosity reduction effect of the composite viscous-reducing system is better than when the dilution ratio of light crude oil is 1.4 (1838 mPa·s). This finding confirms that when washing oil replaces some light crude oil as a thinning medium, a small amount of washing oil/light crude oil has a good viscosity-reducing effect and a low cost.

Table 4 shows that when the dilution ratio of light crude oil increases from 0.8 to 1.2, the viscosity of the mixed oil after dilution decreases from 5193 to 2101 mPa·s at 50 °C. When 3–10% washing oil is added to the light crude oil, the viscosity of the system is reduced, and the viscosity-reducing efficiency is improved. When the dilution ratio of light crude oil is 0.8, and the amount of washing oil is 0 or 10%, the viscosity of the mixed oil after dilution is 5193 and 1838 mPa·s, respectively, and the viscosity reduction rate is increased from 99.00% to 99.65%. These results demonstrate that adding washing oil to light crude oil improves the fluidity of extra-heavy oil and viscosity reduction efficiency; however, the amount of added light crude oil is relatively high (>0.8), resulting in a high cost.

These results show that the washing oil/light crude oil system is an efficient dilute viscosity-reducing agent for extra-heavy oil when washing oil replaces some dilute oil as a blending medium.

### 2.4. Viscosity Reduction Effect of Blending Washing Oil/Aromatic Compound

The aromatic compounds toluene and xylene can be mixed with washing oil. Toluene and xylene are smaller than naphthalene and substituted naphthalene compounds, and theoretically, they can penetrate the interlayer of asphaltene and resin deposits. Thus, a synergistic effect could be achieved by combining them with washing oil.

Like small nails and large nails working together, the washing oil and toluene–xylene penetrate the interior of the asphaltene and resin, break the π-π accumulation, weaken the interaction between the sticky aggregates, and eventually reduce the viscosity of extra-heavy oil. At 50 °C, the viscosities of extra-heavy oil and washing oil are 520,350 and 10 mPa·s, respectively. Table 5 shows the viscosity reduction effect of the washing oil–aromatic compound system on extra-heavy oil.

The dilution ratio of washing oil is 0.2, the viscosity of the mixed oil is 2385 mPa·s, and the viscosity reduction rate is 99.54%. Table 5 shows that the viscosity reduction effect of the washing oil/aromatic compounds (toluene and xylene) on heavy oil is better than that of a single-component washing oil. With the increase in the number of aromatic compounds, the viscosity reduction effect of the multi-component system is better. For example, when washing oil–toluene–extra-heavy oil = 0.14:0.06:1 and washing oil–toluene–heavy oil = 0.12:0.08:1, the viscosities of the mixed oil are reduced to 1763 and 1486 mPa·s, respectively. When washing oil–xylene–extra-heavy oil = 0.14:0.06:1 and washing oil–xylene–extra-heavy oil = 0.12:0.08:1, the viscosity values of the mixed oil are 1714 and 1470 mPa·s, respectively. The viscosity reduction rate of these washing oil–aromatic compounds reaches 99.7%, similar to the effect of toluene and xylene.

These results showed that introducing aromatic compounds into the viscosity-reducing system of washing oil not only reduced the amount of washing oil but also improved the viscosity-reducing effect. However, the price of toluene is 7100~7600 CNY/ton, and the price of xylene is 7200~7900 CNY/ton; they are much more expensive than washing oil. Although the combined washing oil and toluene/xylene system is not very competitive considering the cost, this study provides a new perspective on the development of multi-component viscosity reduction systems for extra-heavy oil.

### 2.5. Viscosity Reduction Effect of Blending Washing Oil and Surfactant

Viscosity reduction using a surfactant is another approach for heavy oil recovery [23,24]. An aqueous solution of a certain concentration is prepared by using a surfactant dissolved in water and then adding it to heavy oil. Under proper temperature and mechanical shear mixing, the crude oil is dispersed in water to form an O/W-type emulsion to achieve emulsification and viscosity reduction [25]. When emulsifying, a viscosity reducer is added to extra-heavy oil, and the friction between oil films is changed to that which occurs between water films under the action of surfactant components. This increases the fluidity of heavy oil and reduces viscosity. In this study, two widely used surfactants were selected: anionic surfactant SDBS and non-ionic surfactant OP-10. We investigated the extra-heavy oil viscosity reduction effect of a multi-component system with different surfactant concentrations (SDBS or OP-10), where washing oil–surfactant solution–extra-heavy oil = 0.16:0.2:1. Table 6 shows that the effect was better with the increased surfactant concentration, and the type of surfactant had a significant influence.

At 50 °C, the viscosity values of extra-heavy oil and washing oil are 520,350 mPa·s and less than 10 mPa·s, respectively. The viscosity reduction effect of a single-component washing oil is as follows: When the dilution ratios are 0.2 and 0.25, the viscosity values of the mixed oil are 2385 and 1214 mPa·s, respectively (Figure 2, Table 2). Table 6 shows that when washing oil–4% SDBS solution–extra-heavy oil = 0.16:0.2:1, the viscosity of the mixed oil is 1541 mPa·s, and the viscosity reduction rate is 99.70%. The multi-component washing oil–surfactant (4% SDBS) viscosity reduction system uses less washing oil (dilution ratio 0.16) to achieve a viscosity reduction effect similar to that of a single-component washing oil (dilution ratio 0.2).

Table 6 shows that the multi-component viscosity reduction system obtained by replacing part of the washing oil with a 2–4% OP-10 solution can reduce the viscosity of extra-heavy oil. The viscosity of the mixture decreased with the increase in OP-10 concentration, and the viscosity reduction rate was greater than 98.56%. However, the viscosity reduction effect of the washing oil/OP-10 system is worse than that of the single-component washing oil. This may be because the non-ionic surfactant OP-10 solution has poor compatibility with the washing oil.

The washing oil–SDBS viscosity reduction system showed better viscosity reduction performance on heavy oil than the washing oil–OP-10 viscosity reduction system. This is because the polar groups in SDBS molecules easily form stronger hydrogen bonds with the polar groups in asphaltene and resin. Under the synergic action of washing oil, they penetrate and disperse throughout the layer molecules of asphaltene and resin. The structure of asphaltene and resin loosens, their spatial structure is destroyed, and the viscosity reduction effect is better. Anionic surfactants are cheap and easy to obtain. Therefore, washing oil–SDBS (washing oil–4% SDBS–extra-heavy oil = 0.16:0.2:1) is a synergistic, multi-component, multi-functional group, high-efficiency viscosity reduction system with a significant application value and development prospects in the extra-heavy oil recovery process.

### 2.6. Comparison of Viscosity Reduction Effects of Different Dilute Media

Diesel oil is commonly used as a dilute viscosity-reducing agent for extra-heavy oil, and it usually has a good viscosity-reducing effect. Table 7 compares the viscosity reduction effects of different dilute media. At 50 °C, the viscosity values of diesel oil with a dilution ratio of 0.4 and washing oil with a ratio of 0.25 after dilution are 1861 and 1214 mPa·s, respectively. This requires less dilute washing oil and provides a better viscosity reduction effect. In addition, the price of washing oil is lower than that of diesel. Therefore, washing oil is an excellent viscosity-reducing medium, and it can replace diesel oil.

Table 7 shows the multi-component viscosity reduction system obtained by reducing the amount of washing oil and mixing a small amount of another dilute medium, including dilute oil, aromatic compounds (toluene/xylene), and 4% SDBS surfactant. The system showed excellent viscosity reduction effects on extra-heavy oil. When washing oil–light crude oil–extra-heavy oil = 0.2:0.1:1, washing oil–aromatic compounds–extra-heavy oil = 0.12:0.08:1, and washing oil–4% SDBS solution– extra-heavy oil = 0.16:0.2:1, the mixed oil viscosity becomes 1470~1775 mPa·s; the viscosity reduction rate is higher than 99.66%. Therefore, the system uses less dilution medium (dilution ratio, 0.2~0.36) and can achieve a viscosity reduction effect similar to that of single-component diesel oil (dilution ratio 0.4).

The viscosity data presented in Table 1, Table 2, Table 3, Table 4, Table 5, Table 6 and Table 7 represent the average values from at least seven independent measurements. The high reproducibility of the experimental results confirms that the differences in viscosity reduction rates between different systems are statistically significant. This effectively eliminates the possibility of measurement saturation or artifacts.

The results show that the system using washing oil as the main mixing medium has a better viscosity reduction effect than light crude oil and diesel oil. The system maintains stability with no observable phase separation or decantation over 7 days. Future studies will systematically investigate the thermal sensitivity of these viscosity reduction systems across a wider temperature range to fully characterize their performance under varying reservoir and pipeline conditions.

### 2.7. Viscosity Reduction Mechanism

**Figure 6:**
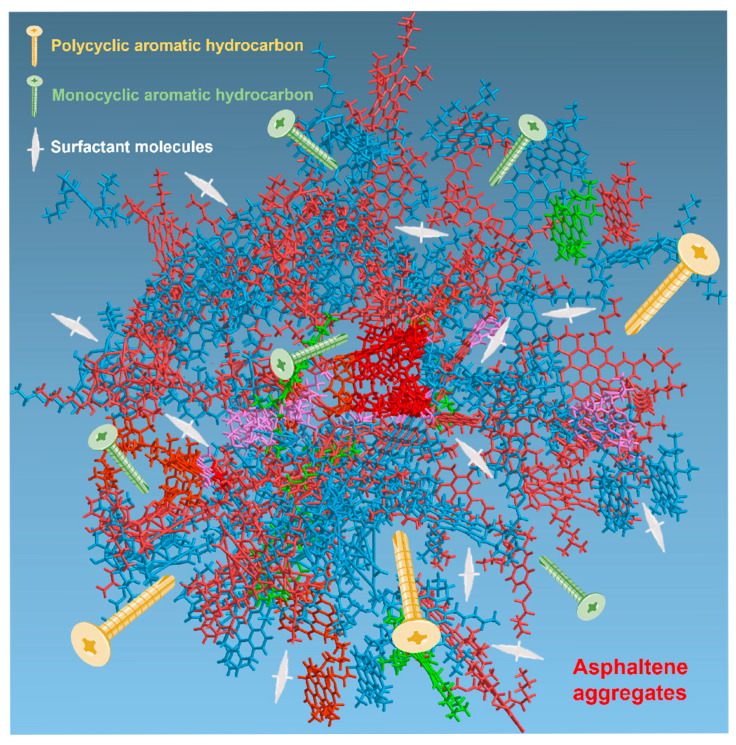
Viscosity reduction mechanism of multi-component viscosity reducer.

Washing oil is rich in aromatic components, like small nails and large nails working together. The washing oil and toluene/xylene penetrate the interior of the asphaltene and resin, break the π-π accumulation, weaken the interaction between the sticky aggregates, and eventually reduce the viscosity of extra-heavy oil. When washing oil is combined with other components, a multi-component and multi-functional synergistic system can be established for the efficient viscosity reduction of extra-heavy oil. The proposed mechanism is shown in Figure 6.

For extra-heavy oil with asphaltene and resin deposits, the components of a viscosity reduction system are like nails acting on a thick wall [26,27,28]. Aromatic components with two or more aromatic rings can only be inserted into areas with relatively large gaps, while monocyclic aromatic components, such as toluene and xylene, can be inserted into areas with smaller gaps. Surfactant molecules can also help, shuttling around inside the extra-heavy oil structure and inserting themselves into cracks of various sizes. Through their combined action, the dense structure of asphaltene and resin is opened and loosened, such that each component can penetrate the aggregate more easily, eventually disperse, and dissolve the asphaltene and resin. In summary, when a variety of components is carefully chosen and combined, they work together and perform their duties to effectively break down extra-heavy oil.

## 3. Materials and Methods

### 3.1. Materials

Extra-heavy oil and light crude oil were obtained from the Tahe Oilfield, Kuche, Xinjiang, China.The extra-heavy oil was sampled from a reservoir depth of 6300–6500 m in a specific block of the oilfield. Prior testing confirmed that its viscosity reaches 520,350 mPa·s at 50 °C, with 22.5% asphaltene content (mass fraction). This aligns with the typical characteristics of extra heavy oil. The light crude oil, taken from the same oilfield, had a viscosity of 220 mPa·s and a density of 0.89 g/cm^3^ at 50 °C. Coal tar was purchased from Guangdong Wengjiang Reagent Co., Ltd. (Shaoguan, Guangdong, China). Washing oil was obtained from Sinopec Northwest China Petroleum Bureau (Urumqi, Xinjiang, China). Toluene was purchased from Chengdu Chron Chemicals Co., Ltd. (Chengdu, Sichuan, China). Xylene and OP-10 were purchased from Shanghai Aladdin Biochemical Technology Co., Ltd. (Shanghai, China). SDBS was obtained from Shanghai Meryer Biochemical Technology Co., Ltd., (Shanghai, China).

**Figure 7:**
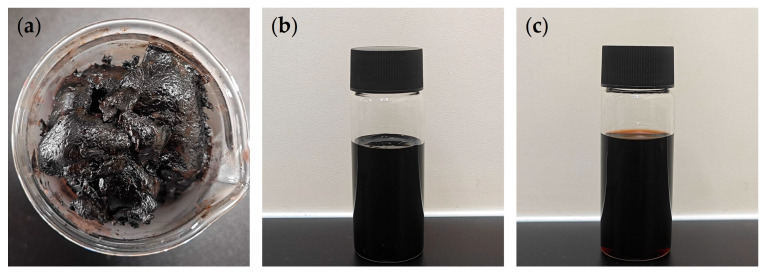
Photos of extra-heavy oil, coal tar, and washing oil: (**a**) extra-heavy oil; (**b**) coal tar; (**c**) washing oil.

Extra-heavy oil is a black semi-solid, coal tar is a black viscous liquid, and washing oil is a yellowish-brown oily liquid (Figure 7).

The viscosity values of coal tar and washing oil at 50 °C are 1834 and 10 mPa·s, respectively. Table 8 and Table 9 show the basic properties of coal tar and washing oil. The data were provided by the supplier and determined according to standard ASTM test methods.

### 3.2. Experiment on Blending and Viscosity Reduction of Extra-Heavy Oil

Heavy oil sample pretreatment: To eliminate the effects of thermal and shear history on the heavy oil, the samples were placed in aging reactors and subjected to sealed rolling at 80 °C (50 rpm) for 4 h using a high-temperature rolling oven (HTD-GL6, Qingdao Hengtaida Electromechanical Equipment Co., Ltd., Qingdao, Shandong, China). The samples were then naturally cooled to room temperature and left to stand for 48 h to achieve a homogeneous and stable state. The pretreated oil samples were subsequently used for further experiments. To prevent solvent volatilization or degradation during heating, all mixing and heating procedures were conducted in sealed containers.

Preparation of surfactant solution: Surfactant solutions were prepared by dissolving weighed surfactants in deionized water according to the specified mass concentrations, followed by stirring until complete dissolution for subsequent use.

Preparation of diluted viscosity reduction mixtures: Based on a predetermined diluent-to-extra-heavy oil mass ratio (dilution ratio), measured amounts of diluent and extra-heavy oil were loaded into aging reactors. The mixtures were subjected to sealed rolling at 80 °C (100 rpm) for 4 h. Pre-experimental tests confirmed that this procedure ensured homogeneous mixing of the diluent and extra-heavy oil under these conditions. The temperature was then reduced to 50 °C and maintained for 2 h prior to viscosity measurement.

Viscosity measurement of mixtures: The samples were removed and stirred again to ensure uniformity. Viscosity testing was performed using an NDJ-5S digital viscometer (Shanghai Jingtian Electronic Instrument Co., Ltd., Shanghai, China; instrument accuracy: ±1%) with rotors No. 3 and No. 4 at rotational speeds of 30 and 60 rpm. The reported viscosity values represent the average of at least three independent measurements. The viscosity of all samples in this study was measured at 50 °C (the pipeline transportation temperature in the oilfield). The viscosity reduction rate of extra-heavy oil after dilution is shown in Formula (1):

η = (I_0_ − I_1_)/I_0_ × 100%, (1)

where η is the viscosity reduction rate of extra-heavy oil after dilution, %; I_0_ is the viscosity of extra-heavy oil before blending viscosity reducer, mPa·s; and I_1_ is the viscosity of mixed oil after blending viscosity reducer, mPa·s.

Optimal dilution point: The optimal dilution point was defined as the diluent-to-extra-heavy oil mass ratio required to reduce the viscosity of the diluted oil to approximately 2000 mPa·s at 50 °C, which meets the viscosity specification for field extraction. All proportions and dilution ratios mentioned in this study are mass-based (*w*/*w*), unless otherwise stated.

### 3.3. Characterization

The chemical composition of the sample was determined via GC-MS analysis, using the GCMS-QP2010SE instrument (Shimadzu, Kyoto, Japan), fitted with a fused silica Equity-5 capillary column (30 m × 0.25 mm; film thickness, 0.25 µm; Supelco, Bellefonte, PA, USA). Helium served as the carrier gas, with a constant flow rate of 1 mL/min, and the injector temperature was maintained at 300 °C. The column temperature program was as follows: an initial hold at 40 °C for 5 min, followed by a linear ramp-up to 300 °C at 15 °C/min, and a final hold at 300 °C for 20 min. Sample introduction was performed via split injection (split ratio, 20:1) with manual injection of 1.0 µL. For the mass spectrometric detection, the ionization energy was set to 70 eV, the ion source temperature was 250 °C, and data were acquired in scan mode over a mass range of 30–650 amu at a sampling rate of 0.5 scan/s. The chemical bonds and functional groups of the sample were analyzed using the Bruker INVENIO-R Fourier-transform infrared (FT-IR) spectrum (Bruker, Ettlingen, Germany), with the attenuated total reflection (ATR) method. Each spectrum was collected as an average of 32 scans between 400 and 4000 cm^−1^ with a 4 cm^−1^ spectral resolution. Spectra were linear-baseline-corrected and normalized.

## 4. Conclusions

Using a by-product of the coal chemical industry as a viscosity-reducing agent for extra-heavy oil is a promising approach. Our study is the very first example of the design and development of a multi-component and multi-functional synergistic system based on oil washing for the efficient viscosity reduction of extra-heavy oil.

The results demonstrate that washing oil (a coal chemical by-product) functions as a highly efficient dilutive viscosity reducer for extra-heavy oil. When formulated with supplementary components, the system enables a significant dosage reduction in washing oil while exhibiting superior viscosity reduction efficacy compared with single-component light crude oil or diesel. This enhanced performance arises from the synergistic interaction between washing oil-derived aromatic hydrocarbons, monosubstituted benzenes (e.g., toluene and xylene), and surfactants. Each constituent fulfills distinct roles. These roles include disrupting π-π stacking in asphaltene networks and facilitating molecular penetration. Together, these actions collectively optimize viscosity reduction efficiency.

The multi-component and multi-function cooperative viscosity reduction system constructed in this study requires little dilution, has a good viscosity reduction effect, and is low-cost. It can effectively reduce the viscosity of extra-heavy oil and provide technical support for efficient viscosity reduction in the Tahe Oilfield of China. It can also be extended to other high-viscosity crude oil production areas.

## Figures and Tables

**Table 1:** Viscosity reduction effect of coal tar on extra-heavy oil (50 °C).

	Extra-Heavy Oil	Coal Tar	Coal Tar–Extra-Heavy Oil (Mass Ratio)	
			2	5
Viscosity/mPa·s	520,350 ± 1381	1834 ± 55	4283 ± 119	1900 ± 41
Viscosity reduction rate/%	-	-	99.18	99.63

**Table 2:** Viscosity reduction effect of washing oil on extra-heavy oil (50 °C).

	Extra-Heavy Oil	Washing Oil	Washing Oil–Extra-Heavy Oil (Mass Ratio)			
			0.15	0.2	0.25	0.3
Viscosity/mPa·s	520,350 ± 1381	<10	5128 ± 107	2385 ± 36	1214 ± 27	622 ± 19
Viscosity reduction rate/%	-	-	99.01	99.54	99.77	99.99

**Table 3:** Viscosity reduction effect of washing oil (as blending medium)/light crude oil on extra-heavy oil (50 °C).

	Extra-Heavy Oil	Washing Oil	Light Crude Oil	Blending Medium (Washing Oil–Light Crude Oil)–Extra-Heavy Oil (Mass Ratio)						
				1.6	1.4	0.7	0.55	0.45	0.4	0.3
				(0:1.6)	(0:1.4)	(0.1:0.6)	(0.15:0.4)	(0.15:0.3)	(0.2:0.2)	(0.2:0.1)
Viscosity/mPa·s	520,350 ± 1381	<10	220 ± 5	1556 ± 26	1838 ± 34	2097 ± 48	1409 ± 27	1974 ± 34	1486 ± 20	1775 ± 13
Viscosity reduction rate/%	-	-	-	99.70	99.65	99.60	99.73	99.62	99.71	99.66

**Table 4:** Viscosity reduction effect of washing oil (as an additive)/light crude oil on extra-heavy oil (50 °C).

	Extra-Heavy Oil	Washing Oil	Light Crude Oil	Blending Medium (Light Crude Oil)–Extra-Heavy Oil (Mass Ratio)							
				1.2		1			0.8		
Additive amount of washing oil/%	-	-	-	0	3	0	3	5	0	5	10
Viscosity/mPa·s	520,350 ± 1381	<10	220 ± 5	2101 ± 39	1572 ± 23	2742 ± 29	2523 ± 42	1619 ± 22	5193 ± 103	3841 ± 75	1838 ± 28
Viscosity reduction rate/%	-	-	-	99.60	99.70	99.47	99.52	99.69	99.00	99.26	99.65

**Table 5:** Viscosity reduction effect of washing oil–aromatic compounds (toluene, xylene) on extra-heavy oil (50 °C).

Washing Oil–Aromatic Compounds–Extra-Heavy Oil (Mass Ratio)	Toluene		Xylene	
	Viscosity/mPa·s	Viscosity Reduction Rate/%	Viscosity/mPa·s	Viscosity Reduction Rate/%
Extra-heavy oil (0:0:1)	520,350 ± 1381	-	520,350 ± 1381	-
Washing oil (1:0:0)	<10	-	<10	-
0.2:0:1	2385 ± 36	99.54	2385 ± 36	99.54
0.16:0.04:1	2324 ± 38	99.55	2350 ± 61	99.55
0.14:0.06:1	1763 ± 33	99.66	1714 ± 34	99.67
0.12:0.08:1	1486 ± 23	99.71	1470 ± 19	99.72

**Table 6:** Viscosity reduction effect of washing oil–surfactant (SDBS, OP-10) on extra-heavy oil (50 °C).

Surfactant Solution Concentration	SDBS		OP-10	
	Viscosity /mPa·s	Viscosity Reduction Rate/%	Viscosity /mPa·s	Viscosity Reduction Rate/%
2%	4111 ± 125.	99.21	7510 ± 128	98.56
3%	2689 ± 49	99.48	6185 ± 89	98.81
4%	1541 ± 23	99.70	5133 ± 128	99.01

Washing oil—surfactant solution–extra-heavy oil = 0.16:0.2:1.

**Table 7:** Viscosity reduction effect of different dilute media on extra-heavy oil (50 °C).

Blending Medium–Extra-Heavy Oil (Mass Ratio)	Washing Oil–Another Medium–Extra-Heavy Oil (Mass Ratio)	Viscosity/mPa·s	Viscosity Reduction Rate/%
0.4	0:0.4 (diesel oil):1	1861 ± 29	>99.62
0.25	0.25:0:1	1214 ± 27	99.77
0.3	0.2:0.1 (light crude oil):1	1775 ± 13	99.66
0.2	0.12:0.08 (toluene):1	1486 ± 23	99.71
0.2	0.12:0.08 (xylene):1	1470 ± 19	99.72
0.36	0.16:0.2 (4% SDBS):1	1541 ± 23	99.70

**Table 8:** Basic physical and chemical properties of coal tar.

Items	Values
Naphthalene content	12.1%
Toluene insoluble content	12.1%
Quinoline insoluble content	3.1%
Water content	2.8%
Ash content	0.06%
Density	1.2998 g·cm^−3^
Carbon residue	13.58%
Condensation point	−6 °C

**Table 9:** Basic physical and chemical properties of washing oil.

Items	Values
Organochlorine content	0.0055%
Naphthalene content	1.03%
Phenol content	0.6%
Water content	0.9%
Density	1.056 g·cm^−3^
Open cup flash point	75 °C
Initial boiling point	193 °C
Distillate before 200 °C (volume fraction)	17%
Distillate before 220 °C (volume fraction)	67%
Distillate before 257 °C (volume fraction)	93%

## Data Availability

The data presented in this study are available upon request from the corresponding author.

## Supplementary Materials

The following supporting information can be downloaded from https://www.mdpi.com/article/10.3390/molecules30224446/s1: Table S1. Qualitative results for total ion chromatographic peak of washing oil.
