# Supplementary material for: A Multi-Component and Multi-Functional Synergistic System for Efficient Viscosity Reduction of Extra-Heavy Oil
Source: Molecules. 2025 Nov 18;30(22):4446. doi: 10.3390/molecules30224446 (PMC12655091; doi:10.3390/molecules30224446)
Supplement: Supplementary file 1 [file molecules-30-04446-s001.zip › molecules-3923723-supplementary.pdf]

molecules

MDPI

Supporting information

A multi-component and multi-functional synergistic system for efficient viscosity reduction of extra heavy oil

Zuguo Yang [1,3,4,†], Yanxia Liu [2,†], Jing Jiang [2,†], Lijuan Pan [1,3], Dandi Wei [1,3], Xingen Feng [1,3], Long He [1,3,*], Jixiang Guo [4,*] and Yagang Zhang [2,*]

[1] Petroleum Engineering Technology Research Institute, Northwest Oilfield Company, Sinopec, Urumqi 830011, China; yangzg.xbsj@sinopec.com (Z.Y.); panlij.xbsj@sinopec.com (L.P.); weidd6099.xbsj@sinopec.com (D.W.); fengxg9687.xbsj@sinopec.com (X.F.)

[2] School of Materials and Energy, University of Electronic Science and Technology of China, Chengdu 611731, China; liuyanxia100@uestc.edu.cn (Y.L.); 202321030310@std.uestc.edu.cn (J.J.)

[3] Key Laboratory of Enhanced Recovery for Fracture-cave Oil Reservoir, Sinopec, Urumqi 830011, China

[4] Unconventional Petroleum Research Institute, China University of Petroleum, Beijing 102249, China

* Correspondence: hel.xbsj@sinopec.com (L.H.); guojx@cup.edu.cn (J.G.); ygzhang@uestc.edu.cn (Y.Z.); Tel.: +86-28-61831516 (Y.Z.)

† These authors contributed equally to this work.

Table S1. Qualitative results of total ions chromatographic peak of washing oil.

	Compound Name	Retention Time /mins	Peak area	Content /%
1	Indane	10.96	19193642.03	0.12
2	Naphthalene	12.83	296854964.91	1.93
3	Quinoline	13.35	359903944.40	2.33
4	Isoquinoline	13.61	91319307.61	0.59
5	1-Methylnaphthalene	13.93	3163058449.46	20.52
6	2- Methylnaphthalene	14.09	1741857190.66	11.30
7	6-Methylquinoline	14.48	80359323.99	0.52
8	Biphenyl	14.70	1031740110.65	6.69
9	2-Ethylnaphthalene	14.85	355306715.46	2.30
10	1,7-Dimethylnaphthalene	14.97	591366226.43	3.84
11	1,8- Dimethylnaphthalene	15.09	959865445.45	6.23
12	1,5 Dimethylnaphthalene	15.28	290626476.04	1.89
13	2,6- Dimethylnaphthalene	15.41	122788536.50	0.80
14	Acenaphthene	15.68	2526044555.75	16.39
15	Naphthalene,2-(1-methylethyl)-	15.83	44767144.55	0.29
16	Benzene, [1-(2,4-cyclopentadien-1-ylidene)ethyl]-	15.89	145127527.79	0.94
17	Dibenzofuran	15.98	1939444436.72	12.58
18	Naphthalene, 1,4,5-trimethyl-	16.18	27050992.84	0.18
19	Naphthalene, 2,3,6-trimethyl-	16.23	27929975.35	0.18
20	Naphthalene, 1,6,7-trimethyl-	16.33	44645205.07	0.29
21	Fluorene	16.54	1144065409.43	7.42
22	Fluorene, 2,4a-dihydro-	16.68	115646339.17	0.75
23	Fluorene, 1,4-dihydro-	16.76	17184584.82	0.11
24	2-Hydroxyfluorene	16.82	65863911.14	0.43
25	9H-Fluoren-9-ol	16.98	83815765.82	0.54
26	Bifenthrin	17.27	11529215.12	0.07
27	9H-Fluorene, 1-methyl-	17.49	17535062.64	0.11
28	Phenanthrene	18.18	27000840.67	0.18
29	Ascorbyl Palmitate	22.30	30114978.67	0.20
30	Acenaphthene	25.23	9461922.44	0.06
31	Naphthalene, 2-ethenyl-	25.31	19199582.55	0.12
32	Benzene, (2,4-cyclopentadien-1-ylidenemethyl)-	25.68	14823798.66	0.10
